# Case Report: When infection wears a rheumatic mask: probable systemic brucellosis mimicking seronegative inflammatory arthritis with sicca symptoms and suspected cervical spondylitis

**DOI:** 10.3389/fmed.2026.1996243

**Published:** 2026-10-27

**Authors:** Yadan Zou, Jing Zhang, Xiaoying Zhang, Qiaolin Zhang, Lina Zhang, Ruohan Yu, Ting Long, Ji Li, Yanfeng Zhang, Sheng-Guang Li

**Affiliations:** 1 Department of Rheumatology and Immunology, Peking University International Hospital, Beijing, China; 2 Department of Pathology, Peking University International Hospital, Beijing, China; 3 Peking University Health Science Center, Beijing, China

**Keywords:** brucellosis, cervical spondylitis, granulomatous lymphadenitis, seronegative inflammatory arthritis, sicca symptoms, zoonotic infection

## Abstract

**Background:**

Brucellosis is a zoonotic infection with protean manifestations. Osteoarticular disease is common, but presentation as seronegative inflammatory arthritis with sicca symptoms, generalized lymphadenopathy, and cervical spondylitis may be mistaken for autoimmune rheumatic disease.

**Case presentation:**

A 58-year-old male cattle farmer presented with intermittent chills, sicca symptoms, symmetric polyarthritis, weight loss, anemia, hypoalbuminemia, and elevated inflammatory markers. Autoimmune serology was negative. Ultrasonography confirmed synovial hyperplasia and tenosynovitis. 18F-FDG PET/CT showed generalized lymphadenopathy and focal C5 uptake; MRI showed C5 marrow edema. Lymph-node biopsy demonstrated non-necrotizing granulomatous inflammation with negative acid-fast and periodic acid-Schiff stains. Positive Rose Bengal and Brucella IgG tests, a standard tube agglutination titer of 1:100, and the clinical context prompted a working diagnosis of probable systemic brucellosis. Blood and bone marrow cultures were negative; paired serology was unavailable. At telephone follow-up, the patient reported taking outpatient rifampicin and doxycycline for 2 years, near-resolution of cervical pain at approximately 3 months, and normalization of inflammatory markers and hemoglobin after more than 6 months. A negative local agglutination result reported by his daughter at approximately 3 months could not be verified. Intermittent bilateral hip and knee pain persisted, requiring as-needed etoricoxib and, at the latest follow-up, a walking aid. Treatment had been extended because symptoms persisted, but the cause of the functional impairment remained undetermined.

**Conclusion:**

Probable systemic brucellosis merits consideration in exposed patients with seronegative inflammatory arthritis, sicca symptoms, and granulomatous lymphadenopathy. Occupational seropositivity, incomplete microbiological confirmation, and unverified follow-up testing limit diagnostic certainty. Symptomatic improvement during antibiotics does not establish the etiology or microbiological cure, and persistent disability warrants reassessment for infection, structural disease, and coexisting rheumatic pathology.

## Introduction

Brucellosis is a systemic zoonotic infection caused by Brucella species and remains an important diagnostic challenge in regions where livestock exposure is common (1, 2). Human disease may involve fever, constitutional symptoms, lymphadenopathy, hepatosplenic disease, and focal complications (2–4). Osteoarticular involvement is among the most frequent focal manifestations, usually presenting as sacroiliitis, spondylitis, or peripheral arthritis (4–6).

Infectious diseases should be included in the differential diagnosis of patients presenting with inflammatory articular manifestations, particularly when exposure history or systemic features raise concern. Inflammatory polyarthritis, synovitis, high inflammatory markers, sicca symptoms, and generalized lymphadenopathy may overlap with seronegative rheumatoid arthritis, Sjogren-like disease, sarcoidosis, lymphoma, or tuberculosis. Accurate diagnosis requires integration of exposure history, targeted serological testing, culture when available, imaging, pathology, and longitudinal assessment (7–10).

This report describes probable systemic brucellosis in a cattle farmer presenting with seronegative inflammatory arthritis, sicca symptoms, granulomatous lymphadenopathy, and suspected cervical spondylitis. For rheumatologists, the case highlights two practical issues: treatable infection should be evaluated before immunosuppressive therapy is escalated in patients with compatible exposure and systemic red flags, and improvement in inflammatory markers does not establish the cause or resolution of long-term musculoskeletal disability.

## Case description

A 58-year-old male cattle farmer was admitted to the rheumatology department because of intermittent chills for 7 months and joint swelling and pain for 4 months. Four months before admission, he developed dry mouth requiring water to swallow dry food, dry eyes, nocturia, and shoulder and knee pain. Initial outside testing showed an erythrocyte sedimentation rate (ESR) of 54 mm/h and C-reactive protein (CRP) of 106.8 mg/L; rheumatoid factor and anti-cyclic citrullinated peptide antibody were not elevated. Two months before admission, symmetric pain and swelling involved the wrists, metacarpophalangeal joints, proximal interphalangeal joints, elbows, shoulders, knees, ankles, and feet, together with fatigue, anorexia, weight loss, evening chills, and sweating after warming. The relative clinical timeline is shown in Table 1.

**Table 1:** Relative clinical diagnostic timeline.

Relative time	Clinical findings/actions	Recorded result or status
7 months before admission	Intermittent chills began.	No defined diagnosis was recorded at symptom onset.
4 months before admission	Dry mouth, dry eyes, nocturia, shoulder and knee pain; ESR 54 mm/h, CRP 106.8 mg/L; rheumatoid factor and anti-CCP antibody not elevated.	Sicca symptoms and inflammatory joint symptoms were present.
2 months before admission	Progressive symmetric polyarthritis, fatigue, anorexia, weight loss, evening chills, and sweating after warming.	Symptoms involved both joints and systemic features.
At admission (day 0)	CRP 205.88 mg/L, ESR 75 mm/h, hemoglobin 77 g/L, albumin 27.5 g/L; generalized lymphadenopathy and synovitis.	Infection, tuberculosis, lymphoma, sarcoidosis, and seronegative autoimmune rheumatic disease were included in the differential diagnosis.
Hospital days 2–9	Autoimmune serology negative; T-SPOT negative; PET/CT showed FDG-avid lymphadenopathy and C5 uptake; lymph-node biopsy showed non-necrotizing granulomatous inflammation.	Pathology described reactive lymphoid hyperplasia with granulomatous inflammation.
Hospital days 10–14	Rose Bengal plate agglutination positive, Brucella IgG positive, standard tube agglutination test 1:100; cervical MRI showed C5 marrow edema.	Probable systemic brucellosis with suspected cervical spondylitis; final blood and bone marrow cultures negative.
After discharge, about 3 months	Cervical pain reportedly nearly resolved. The daughter reported a negative local agglutination test, recorded by the clinician; the original report was unavailable.	Clinical improvement was reported; the serology result was unverified and did not establish cure.
After treatment for more than 6 months	Patient reported normalization of ESR and CRP and hemoglobin recovery from approximately 78 g/L to the normal range; original follow-up reports unavailable.	Patient-reported laboratory improvement.
After discharge, up to 2 years	Oral rifampicin and doxycycline were reportedly continued because symptoms had not completely resolved. No adverse effects requiring intervention were reported.	Objective reassessments and systematic safety-monitoring records were unavailable.
From 2023 to most recent follow-up	Intermittent bilateral hip and knee pain persisted; oral etoricoxib was taken as needed.	Residual lower-limb joint pain persisted.
Most recent follow-up	Walking-aid dependence was reported; the patient could not recall its onset. Repeat imaging and objective functional assessment were unavailable.	Major adverse functional outcome of undetermined cause.

His history was notable for long-term occupational cattle exposure. He could not recall whether he had consumed unpasteurized dairy products. Residence-related endemic risk, livestock infection status, and screening of family members or close contacts were not documented. He had no known tuberculosis history or close tuberculosis contact. An outside echocardiogram performed before admission showed left atrial enlargement, mild mitral regurgitation, increased aortic valve flow velocity consistent with a hyperdynamic state, and trace pericardial effusion; no vegetation was reported. On admission, he was afebrile, with a blood pressure of 143/70 mmHg and a pulse of 55 beats per minute. Physical examination showed conjunctival pallor, reduced breath sounds at both lung bases, bilateral lower-limb pitting edema, swelling and tenderness of multiple joints, restricted shoulder and knee motion, and multiple enlarged cervical, axillary, and inguinal lymph nodes. No heart murmur was documented.

Laboratory evaluation showed systemic inflammation and inflammatory anemia: CRP 205.88 mg/L, ESR 75 mm/h, hemoglobin 77 g/L, platelet count 489 × 10^^9^/L, albumin 27.5 g/L, fibrin degradation products 27.06 microg/mL, and D-dimer 3,601 ng/mL. Procalcitonin was 0.09 ng/mL. Urinary beta2-microglobulin was 9.97 mg/L, urinary N-acetyl-beta-D-glucosaminidase was 45.3 U/L, urinary retinol-binding protein was 3.99 mg/L, and 24-hour urinary protein excretion was 281.51 mg/day.

Autoimmune testing included antinuclear antibody panel, anti-SSA/Ro60, anti-SSA/Ro52, anti-SSB/La, rheumatoid factor, anti-cyclic citrullinated peptide antibody, antineutrophil cytoplasmic antibodies, anti-myeloperoxidase antibody, anti-proteinase 3 antibody, anti-glomerular basement membrane antibody, autoimmune liver antibodies, and anti-endothelial cell antibodies; all were negative. Serum IgG4 was not elevated. The differential diagnosis during admission included infection, tuberculosis, lymphoma, sarcoidosis, and seronegative autoimmune rheumatic disease.

The tuberculosis and opportunistic infection evaluation recorded no cough, sputum production, abdominal pain, diarrhea, dysuria, or urinary frequency. T-SPOT testing was negative, chest CT did not show a typical pulmonary tuberculosis lesion, and cytomegalovirus and Epstein–Barr virus DNA were negative. Lymph-node acid-fast and periodic acid-Schiff stains were later negative.

Musculoskeletal ultrasonography demonstrated synovial hyperplasia and flexor tenosynovitis of the bilateral metacarpophalangeal and selected interphalangeal joints. Hand radiographs showed distal interphalangeal osteoarthritis and periarticular osteopenia; right hip radiographs showed degenerative changes. 18F-FDG PET/CT showed increased uptake around the bilateral shoulders, hips, and sternoclavicular joints. Uptake at several rib-vertebral and facet joints, the L4 posterior elements, and the right sacroiliac region was interpreted in the imaging report as more likely degenerative. Multiple FDG-avid lymph nodes involved the cervical, supraclavicular, axillary, anterior chest wall, iliac, and inguinal regions, with focal uptake in the C5 vertebral body (Figure 1B). Cervical MRI showed mild C5 marrow edema alongside multilevel degenerative changes and disc protrusions (Figure 1A); cervical spondylitis was suspected clinically.

**Figure 1:**
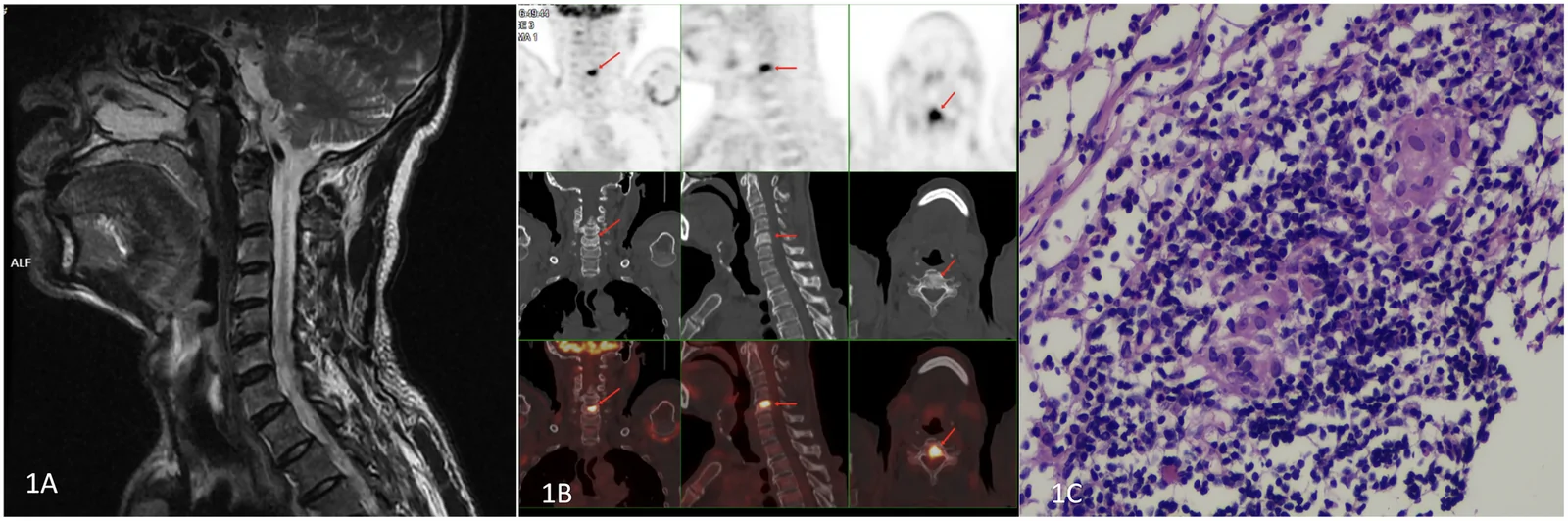
Multimodal findings in the diagnostic workup. **(A)** Cervical MRI showing C5 marrow edema with coexisting degenerative changes; spondylitis was suspected clinically. **(B)** 18F-FDG PET/CT showing focal C5 uptake (red arrows). **(C)** Lymph-node biopsy showing non-necrotizing granulomatous inflammation with plasma cells and focal eosinophilic infiltration. Acid-fast and periodic acid-Schiff stains were negative (not shown). The imaging and histological findings are not specific for Brucella infection.

An ultrasound-guided core biopsy of a right inguinal lymph node showed preserved lymph-node architecture with tissue edema, scattered non-necrotizing granulomatous nodules, locally increased plasma cells, and scattered or focal eosinophilic infiltration (Figure 1C). Immunohistochemistry showed CD3 positivity, follicular CD20 staining, a CD21-positive follicular dendritic-cell network, CD68 positivity, and a Ki-67 index of approximately 30%; the CD138 result was pending in the available report. Acid-fast and periodic acid-Schiff stains were negative. The pathology report described reactive lymphoid hyperplasia with granulomatous inflammation and suggested additional evaluation for special infection. No tissue mycobacterial or fungal culture or molecular test result was available.

Brucellosis testing was performed during admission. Rose Bengal plate agglutination and Brucella IgG were positive, and the standard tube agglutination test was reported positive at 1:100. Brucella IgM, quantitative IgG values, and a short-interval confirmatory serum sample were not documented. Blood and bone marrow cultures were pending at discharge; the final results of both cultures were negative. The discharge diagnosis was systemic brucellosis with inflammatory arthritis, granulomatous lymphadenitis, and suspected cervical spondylitis. With the available evidence, the working diagnosis remains probable systemic brucellosis.

After infectious-disease consultation, the patient was treated with rifampicin 750 mg once daily plus a tetracycline-class agent, documented locally as minocycline 100 mg twice daily, and ceftriaxone 2 g once daily during the early complicated phase for suspected spinal involvement. The planned approach was prolonged oral combination therapy for at least 12 weeks, with ceftriaxone for approximately 4–6 weeks. At telephone follow-up, he reported that outpatient treatment had consisted of oral rifampicin and doxycycline for 2 years. According to the follow-up history, treatment was extended because symptoms had not completely resolved. No adverse effects requiring clinical intervention were reported during treatment. Outpatient doses, treatment interruptions, and systematic laboratory safety-monitoring records were unavailable. Nonsteroidal anti-inflammatory treatment was used for symptomatic arthritis, and glucocorticoids or disease-modifying antirheumatic drugs were not started.

At telephone follow-up, the patient reported near-disappearance of cervical pain after approximately 3 months of anti-Brucella treatment. His daughter reported a negative local agglutination result at approximately 3 months, which the clinician recorded. The original serology report, assay type, numerical titer, and laboratory cutoff were unavailable, and no serial IgG or IgM results were documented. He also reported normalization of ESR and CRP and recovery of hemoglobin from approximately 78 g/L to the normal range after more than 6 months; the original follow-up laboratory reports were unavailable. Intermittent bilateral hip and knee pain persisted from 2023 onward, with as-needed oral etoricoxib. At the most recent follow-up, he required a walking aid. He could not recall when he began using a walking aid. No repeat hip or knee imaging was available, and contemporaneous joint, neurological, and functional assessments were not documented. No new immunosuppressive therapy was reported.

## Discussion

This case is clinically relevant because the initial clinical picture could be read through a rheumatologic lens. The patient had symmetric inflammatory polyarthritis involving small and large joints, sicca symptoms, tubular injury markers, generalized lymphadenopathy, and very high inflammatory markers. These findings can overlap with seronegative rheumatoid arthritis, Sjogren-like disease, sarcoidosis, lymphoma, and tuberculosis. Negative autoimmune serology does not exclude a coexisting rheumatic disease, but occupational cattle exposure and systemic constitutional symptoms warranted infectious-disease assessment before immunosuppression.

Brucellosis is well known for protean manifestations and focal complications (1–4). Osteoarticular disease may involve peripheral joints, sacroiliac joints, or the spine (4–6, 11, 12). Recent reviews emphasize the diagnostic and functional consequences of spinal involvement, including cervical disease (12, 13). Here, synovitis, periarticular uptake, and C5 marrow edema were compatible with an osteoarticular presentation but were not organism-specific. Coexisting degenerative findings and the absence of spinal tissue microbiology limit attribution of these lesions to Brucella.

The diagnostic value of brucellosis serology depends on context. Cross-reactivity, assay variability, and previous exposure can produce positive results without active infection (7–10). Chinese guidance recognizes an agglutination endpoint titer of 1:100, whereas 1:160 is commonly used internationally; this difference helps explain the original clinical interpretation but does not independently prove active infection (14). Occupational exposure, constitutional illness, multisite inflammation, and positive serology justified considering brucellosis and starting treatment. Nevertheless, IgG positivity in a repeatedly exposed farmer may reflect remote exposure. A single 1:100 result without a short-interval repeat or a demonstrated fourfold rise cannot resolve this uncertainty. Both final cultures were negative, which neither confirms nor excludes brucellosis (9, 10). Accordingly, probable systemic brucellosis is a clinical working diagnosis, not a claim of microbiologically confirmed infection.

The reported negative agglutination result at approximately 3 months deserves particular caution. Antibodies often persist for months or years after treatment, although kinetics vary with the assay and patient (8–10). Without the original report, a numerical titer, or confirmation that the same method and cutoff were used, this daughter-reported result, recorded by the clinician, cannot establish true seroreversion. A low starting titer or between-assay differences could influence whether a result is called negative, but neither explanation is demonstrated here. The result is not evidence of microbiological cure and does not retrospectively confirm the original diagnosis.

Endocarditis and tuberculosis were important competing or coexisting considerations. Brucellar endocarditis is uncommon but accounts for a substantial proportion of brucellosis-related mortality (15). No vegetation or murmur was documented, but transesophageal echocardiography was not recorded; endocarditis was neither demonstrated nor definitively excluded. The record therefore provides no documented endocarditis indication for the 2-year course. Negative T-SPOT, absence of typical pulmonary lesions, and negative tissue acid-fast staining do not exclude extrapulmonary tuberculosis.

The lymph-node core showed a reactive lymphoid pattern and non-necrotizing granulomas, not a Brucella-specific lesion. Negative acid-fast and periodic acid-Schiff stains reduce direct histological evidence for mycobacterial or fungal infection but do not exclude either. Sarcoidosis remains a noninfectious alternative, while a limited core biopsy cannot definitively exclude unsampled lymphoma. The absence of tissue culture or molecular confirmation restricts etiological interpretation; plasma cells and focal eosinophils do not resolve this differential.

Uncomplicated brucellosis is commonly treated with combination therapy using doxycycline plus rifampicin or doxycycline plus an aminoglycoside (16–18). Spinal disease usually requires a longer course; contemporary reviews describe regimens commonly lasting 3–6 months, with extension individualized to clinical and radiological findings (11, 12). The inpatient regimen used rifampicin and minocycline with early ceftriaxone for suspected spinal involvement. The reported 2-year outpatient course substantially exceeds usual regimens, including most spinal regimens. The reported reason for extension was incomplete symptom resolution, but persistent pain alone does not demonstrate viable infection. Without objective reassessments or documented relapse, the necessity of this duration cannot be established. It should not be interpreted as a recommended duration. No adverse effects requiring intervention were reported; nevertheless, the absence of systematic safety-monitoring records precludes a comprehensive assessment of long-term tolerability.

Treatment response is also diagnostically nonspecific. Tetracyclines, particularly minocycline, have shown anti-inflammatory effects in rheumatoid arthritis trials (19). Thus, minocycline or doxycycline exposure, together with symptomatic anti-inflammatory treatment, may have attenuated joint symptoms or inflammatory indices independently of bacterial clearance. This possible contribution cannot be quantified in this patient, but it weakens any inference that improvement during antibiotics proves brucellosis or excludes coexisting inflammatory arthritis.

Persistent bilateral hip and knee pain with walking-aid dependence represents a major adverse functional outcome, not simply a minor residual symptom. The patient was 58 years old at initial presentation; the subsequent telephone assessment did not establish when mobility deteriorated. Degenerative right hip changes were already documented at admission and provide one possible contributor, but they do not explain bilateral symptoms or the severity of disability. Structural joint damage, mechanical disease, deconditioning, persistent or relapsing infection, and coexisting seronegative inflammatory arthritis remain alternatives. An infection-triggered inflammatory arthropathy is possible but unproven. Persistent pain alone cannot diagnose chronic active brucellosis, and earlier normalization of inflammatory markers cannot establish current infection status (9, 10, 12). In-person joint and neurological examination, updated inflammatory markers, and targeted hip, knee, or spinal imaging would help distinguish these possibilities; renewed microbiological assessment would be appropriate if active infection were suspected. These investigations were not available, so neither irreversible brucellar damage nor an autoimmune overlap can be asserted.

The immunological mechanisms underlying this presentation remain uncertain. Brucella infection can drive granulomatous inflammation and immune activation, but this case did not include cytokine profiling, flow cytometry, T-cell subset analysis, Brucella PCR from tissue, or tissue immunostaining for immune pathways. Eosinophilic infiltration in the lymph node was a nonspecific histological finding within granulomatous lymphadenitis; its diagnostic value for brucellosis or for distinguishing brucellosis from sarcoidosis cannot be established from this case.

The main strength of this report is the multidisciplinary diagnostic trajectory: rheumatologic presentation, multimodal imaging, lymph-node pathology, and targeted infectious testing. Its limitations include single-case design, negative cultures without microbiological confirmation, absence of paired serology, a modest initial agglutination titer, unverified follow-up laboratory results, symptom-driven treatment extension without documented objective reassessment and incomplete safety monitoring, incomplete objective sicca testing, limited echocardiographic evaluation, and no objective reassessment of long-term disability. The case supports sustained diagnostic vigilance rather than a definitive causal attribution: probable brucellosis should remain in the differential diagnosis of exposed patients, but both the original diagnosis and persistent disability require critical reassessment.

## Reporting guideline statement

This case report follows the CARE reporting guidelines (20).

## Patient perspective

During telephone follow-up, the patient reported taking oral rifampicin and doxycycline for 2 years after discharge and near-disappearance of cervical pain after approximately 3 months. He recalled later normalization of inflammatory markers and hemoglobin. Intermittent bilateral hip and knee pain nevertheless persisted from 2023, requiring as-needed etoricoxib, and he reported needing a walking aid at the latest follow-up.

## Data Availability

The original contributions presented in the study are included in the article, further inquiries can be directed to the corresponding author.
